# Metallo-β Lactamase Producing Non-Fermentative Gram-Negative Bacilli from Various Clinical Isolates in a Tertiary Care Hospital: A Descriptive Cross-sectional Study

**DOI:** 10.31729/jnma.6408

**Published:** 2021-09-30

**Authors:** Mahendra Shrestha, Ratna Baral, Lok Bahadur Shrestha

**Affiliations:** 1 Department of Microbiology & Infectious Diseases, B. P. Koiraia Institute of Health Sciences, Dharan, Sunsari 56700, Nepal; 2 School of Medical Sciences and The Kirby Institute, Faculty of Medicine, University of New South Wales, Sydney, New South Wales, Australia

**Keywords:** *beta-lactamase*, *multidrug resistant*, *Pseudomonas aeruginosa*

## Abstract

**Introduction:**

Non-fermentative gram-negative bacilli are common causes of human infections especially nosocomial infections. These organisms are usually resistant to multiple antimicrobial agents including carbapenems. The study aimed to find out the prevalence of metallo-ß-lactamase producing non-fermentative gram-negative bacilli among the samples which yielded growth of bacteria in a tertiary care hospital.

**Methods:**

This is a descriptive cross-sectional study conducted in a tertiary care hospital from April 2017 to May 2017. Convenience sampling method was used. Bacterial identification, characterization and antimicrobial susceptibility testing were done by following standard microbiological guidelines. MetaUo-|3-lactamase production was detected by using combined disk diffusion test and double-disc synergy test. Data were analyzed by using Statistical Package of Social Science software version 16. Point estimate at 95% confidence interval was calculated along with frequency and proportion for binary data.

**Results:**

Among 628 samples which yielded growth of bacteria, 118 (18.79%) at 95% Confidence Interval (15.74-21.84) were metallo-ß-lactamase producing non-fermentative gram-negative bacilli. Among them, 54 (45.76%) were Pseudomonas aeruginosa and 64 (54.24%) were Acinetobacter baumannii.

**Conclusions:**

A high prevalence of metallo-ß-lactamase production was observed among the nonfermentative gram-negative bacilli than the study done in similar settings. It is mandatory to perform routine monitoring of metallo-ß-lactamase producing isolates in clinical laboratories in order to help the clinicians prescribe proper antibiotics.

## INTRODUCTION

Metallo-β-lactamases (MBL) are a group of enzymes that induce the hydrolysis of a broad set of P-lactam drugs including carbapenems.^1^ Pseudomonas aeruginosa (P. aeruginosa) and Acinetobacter baumannii are the most common gram-negative, non-fermentative bacteria encountered in the laboratory from various clinical specimens. These organisms have characteristics of being resistant to multiple antimicrobial agents including carbapems.^2^ MBL inactivates carbapenems and has hindered the use of these antibiotics.^3,4^

These enzymes are inactivated by chelating agents such as ethylene diamine tetra acetic acid.^1,3,4^ Hence, detection of MBL producing P. aeruginosa and A. baumannii is crucial for optimal treatment of the patient and to reduce the spread of resistance.^5,6^

The study aims to find out the prevalence of metallo-β-lactamase producing non-fermentative gram-negative bacilli among the samples which yielded growth of bacteria in a tertiary care hospital.

## METHODS

This descriptive cross-sectional study was conducted at B. P. Koirala Institute of Health Sciences (BPKIHS) from April 2017 to May 2017. Ethical clearance was obtained from Departmental Research Unit, which is a part of institutional review board of BPKIHS (DRU/Micro/02/017). Convenience sampling technique was used. Samples which yielded growth of bacteria were included and the samples which did not yield the growth of bacteria were excluded.

The sample size was calculated by following formula:

n = Z^2^ × p × q / e^2^

  = (1.96)^2^ × (0.5) × (1 - 0.5) / (0.05)^2^

  = 385

where,

n = minimum required sample size
Z = 1.96 at 95% Confidence Interval (CI)
p = prevalence of metallo-β-lactamase non-fermenter gram-negative bacilli for maximum sample size, 50%
q = 1-p
e = margin of error, 5%

The minimum sample size calculated was 385, however, we took 628 samples which yielded growth of bacteria.

After receiving the clinical samples, they were inoculated onto blood agar and MacConkey agar. Cysteine lactose electrolyte deficient medium was used for urine specimen. The inoculated medium was incubated aerobically at 37°C for 24 hours. In case of blood sample, inoculated was done in brain heart infusion broth and incubated for aerobically 24 hours before subculturing onto blood agar, MacConkey agar, and chocolate agar. The colonies obtained on solid media were subjected for identification and antimicrobial susceptibility. Organisms were identified on the basis of colony characteristics, gram's stain, pigmentation, motility and different biochemical tests like cytochrome oxidase, catalase, urease test, polymyxin susceptibility, nitrate reduction, amino acid decarboxylation tests and sugar fermentation tests as per standard microbiological guidelines.^8^ Antimicrobial susceptibility test was performed on Mueller Hinton agar (MHA) plate by Bauer-Kirby disc diffusion methods following clinical and laboratory standards institute (CLSI) guidelines. The organisms were tested against following antimicrobial discs: amikacin (30μg), gentamycin (10μg), tobramycin (10μg), ciprofloxacin (5μg), imipenem (10μg), aztreonam (30|μg), piperacillin (100μg), piperacillin-tazobactam (100/10μg), ceftazidime (30μg), carbenicillin(100μg) [Hi-Media, India].^9-10^ Zone of inhibition was interpreted based on the guidelines provided by CLSI.^9^ Multidrug resistance (MDR) was defined as resistance to at least one agent in more than three or more classes of antimicrobials as proposed by Magiorakos et al.^11^ The imipenem resistant P. aeruginosa and A. baumannii isolates were screened for MBL production by combined disk diffusion test and double-disc synergy test.^12-14^ Within 15 minutes after adjusting the turbidity of the inoculum suspension, organisms were plated on MHA plate by lawn culture technique. Two imipenem (IMP) discs (10μg), one containing 10μl of 0.5M (750μg) anhydrous ethylenediaminetetraacetic acid (EDTA), were placed 25mm apart and incubated overnight at 37°C. Increment in zone diameter of > 7mm surrounding the IMP-EDTA disk compared to imipenem disk alone was considered as positive.^14,15^ For double disc synergy test, the test organism was inoculated on MHA as above. Two-discs were placed, 20mm apart from center to center, one imipenem (10 μg) disc and another blank disc to which 10μl of 0.5 M EDTA. Enhancement of the zone of inhibition in the area between imipenem and EDTA disc in comparison to zone size on the far side of the drug was considered as a positive result.^12-13^

All the information were entered in Microsoft Excel 2013 and analyzed using Statistical Package for the Social Sciences (SPSS version 11.5) software.

## RESULTS

Among 628 samples which yielded growth of bacteria, 118 (18.79%) were metallo-|3-lactamase producing nonfermentative gram-negative bacilli. Among them, 54 (45.76%) were P. aeruginosa and 64 (54.24%) were A. baumannii. Out of the total 54 clinical isolates of P. aeruginosa, 34 (63%) were from male patients and 20 (37%) from female patients. Likewise, out of the total 64 clinical isolates of A. baumannii, we obtained 42 (66%) from male patients and 22 (34%) from female patients. The maximum number of both organisms were isolated in adult age group (Table 1).

**Table 1:** Distribution of isolates based on age, sex, origin, and specimen.

	Pseudomonas aeruginosa (n = 54)	Acinetobacter baumannii (n = 64)
	n (%)	n (%)
**Sex**		
Male	34 (63)	42 (65.6)
Female	20 (37)	22 (34.4)
**Age**		
Children (<14 years)	5 (9.25)	10 (15.6)
Adults (15-60 years)	33 (61)	41 (64)
Elderly (>60 years)	16 (29.6)	13 (20.31)
**Origin**		
Emergency	3 (5.5)	4 (6.25)
Wards	20 (37)	19 (29.6)
OPD^*^	17 (31.4)	18 (28.12)
ICU^†^	14 (26)	23 (36)
Specimen		
Blood	8 (14.8)	6 (9.37)
ETT^‡^	11 (20.3)	15 (23.43)
Sputum	10 (18.5)	12 (18.75)
Urine	8 (14.8)	8 (12.5)
Swab	5 (9.25)	10 (15.6)
Pus	12 (22.2)	13 (20.31)

* OPD: Outpatient department

† ICU: Intensive care unit

‡ ETT: Endotracheal tube

**Figure 1:**
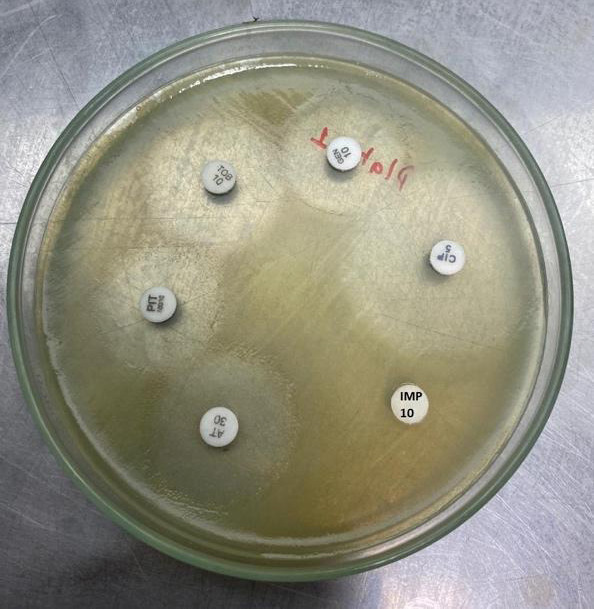
Antimicrobial sensitivity test of P. aeruginosa.

We obtained most P. aeruginosa isolates from wards 20 (37%), followed by OPD 17 (31%), while the most common source of A. baumannii was ICU 23 (36%) followed by wards 19 (30%). Sample wise analysis of the isolates showed that P. aeruginosa was most commonly isolated from the Pus sample 12 (22%), while endotracheal tube 15 (23%) yielded the highest number of A. baumannii. Among Pseudomonas aeruginosa, resistance against aztreonam, amikacin, ceftazidime, ciprofloxacin, imipenem, and piperacillin-tazobactam was found to be 10%, 14%, 37%, 25%, 25%, and 16% respectively. Similarly, among A. baumannii, 20%, 46%, 70%, 65%, 46%, and 48% resistance was observed against aztreonam, amikacin, ceftazidime, ciprofloxacin, imipenem, and piperacillin-tazobactam. Among 27 (44%) of P. aeruginosa isolates and 47 (73.4 %) of A. anitratus were multi-drug resistant (Figure 1).

**Figure 2:**
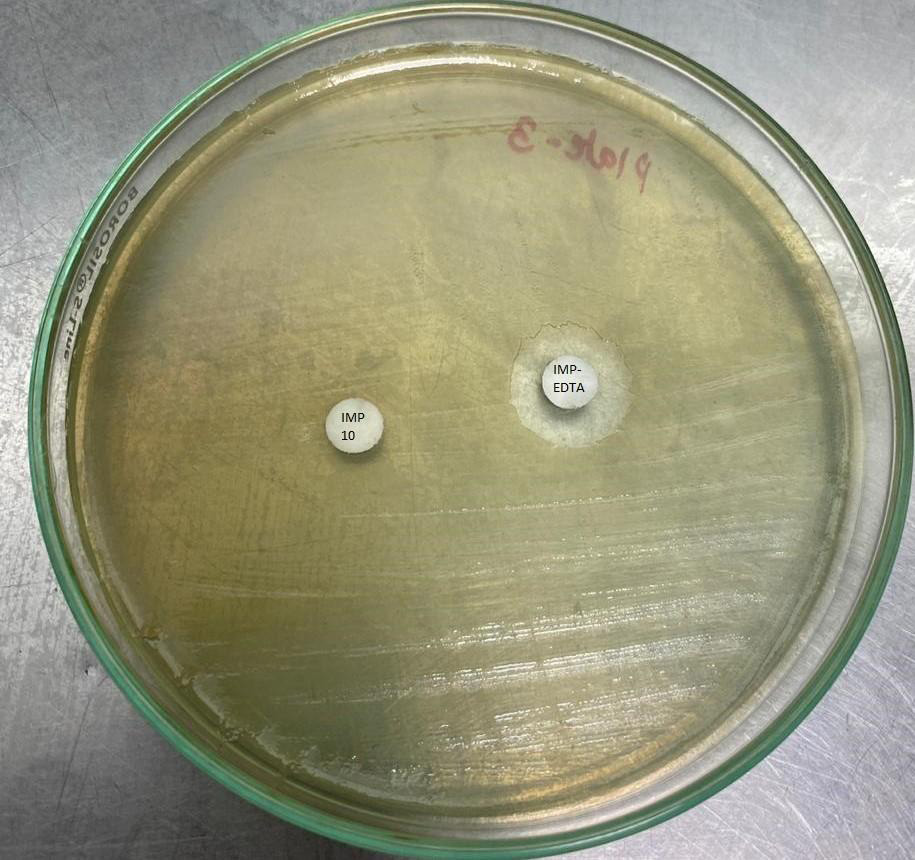
MBL detection by IMP and IMP-EDTA combined disk diffusion method.

Among the total clinical isolates, 14 (11.86%) were imipenem resistant P. aeruginosa and 30 (25.42%) were A. baumannii. We tested these strains for MBL by IMP-EDTA combined inhibition method and double disk synergy test (Figure 2).

MBL producing Pseudomonas aeruginosa was 7 (50%) with IMPEDTA combined inhibition and double disk synergy test. Similarly, MBL producing A. baumannii was 20 (66.67%) with both combined inhibition and double disk synergy test (Table 2).

**Table 2:** Result of MBL producing Pseudomonas aeruginosa by IMP-EDTA combined disk inhibition method and double disk synergy test.

	Total isolates	Imipenem Resistant n (%)	MBL Positive n (%)
Pseudomonas aeruginosa	54	14 (25.9)	7 (50)
Acinetobacter baumannii	64	30 (46.8)	20 (66.67)

## DISCUSSION

Pseudomonas aeruginosa and Acinetobacter baumannii have emerged as the most worrisome pathogens for health-care facilities worldwide. These organisms cause multi-drug resistant nosocomial infections. Production of MBL by P. aeruginosa and A. baumannii has therapeutic consequences since these organisms also carry multi-drug resistance genes. Reporting of MBL will help infection control practitioners in mitigating the spread of these isolates.^1^-^11^-^15^-^16^

In the present study, among 118 non-fermenter GNB, 54 were Pseudomonas aeruginosa and 64 were Acinetobacter baumannii. Among P. aeruginosa isolates, 14 were resistant to imipenem and 7 (50%) of them were MBL producers. Similarly, 20 A. baumannii isolates were imipenem resistant and 20 (66.67%) were MBL producers. The result was supported by similar studies conducted by Azmi et al.^17^ Kazi et al.^18^ Young et al.^13^ and Pitout et al.^19^ Our prevalence of MBL does not correlate with other studies. A lower prevalence of MBL was stated in the study of John et al.^20^ Dumaru et al.^21^ De et al.^22^ Gautam et al.^23^ and Kaur et al.^24^ Another study conducted in Kathmandu, Nepal also reported a low prevalence of MBL among P. aeruginosa (16%) and A. baumannii (22%).^25^ On the other hand, a higher prevalence of MBL production was described by Irfan et al.^27^ and Koirala et al.^27^ in which, 100% & 96.6% and 73.91% and 75%, MBL producers were reported. Similarly, a study conducted in Egypt reported MBL production in 95% of A. baumannii isolates.^28^ The variation might be attributable to the difference in sample size and the fact that our hospital is a tertiary referral center and most patients arriving here have already been treated with multiple antimicrobials elsewhere.

Antimicrobial susceptibility pattern of the isolates showed a variable degree of resistance. On the account of MBL producing P. aeruginosa, 85% were resistant against ceftazidime, piperacillin, piperacillin-tazobactam, and ciprofloxacin, 71% to carbenicillin and 57% were resistance against aztreonam, tobramycin, gentamycin, and amikacin. Similar results were obtained in the study conducted by Dumaru et al.^21^ and Anane et al.^29^ In another study, Mishra et al.^30^ reported 100 % resistance to Piperacillin and ceftazidime each, 83.3% to Imipenem, 50% to gentamycin, 66.67% to ciprofloxacin. Multi-drug resistance was observed among 44% of P. aeruginosa and 73.4% of A. baumannii isolates in our study. The frequency is lower than the study done by Matbainor H et al. who reported MDR in 100% isolates.^33^ A study conducted in Nepal also showed a higher proportion of MDR i.e., 79.3%. in A. anitratus and 84.2% for P. aeruginosa.^31^ However, a study in Ethiopia reported a low degree of multidrug resistance (8.4%) among these organisms.^33^ The higher rates of resistance in the present study reflects the irrational and extensive use of these antibiotics in our medical settings, which resulted in emergence of resistance.

The resistance pattern when compared between MBL producing and non-producing P. aeruginosa showed significant differences. Among MBL positive Acinetobacter baumannii, 90% were resistant against ceftazidime and ciprofloxacin, 85% to piperacillin and carbenicillin and 80% to piperacillin-tazobactam. A study conducted by Anwar et al.^33^ and Kaur et al.^34^ reported a similar pattern of drug resistance. The antimicrobial resistance pattern was compared between MBL producer A. baumannii and non-producers which showed statistically significant differences. The result of our study is similar to Dumaru et al.^21^ and Thapa et al.^6^ Other studies conducted in our hospital also reported a similar antimicrobial resistance pattern in P. aeruginosa and A. baumannii.^35,36^

Thus, the findings of our study suggest that the emergence of MBL producing P. aeruginosa and A. baumannii in our clinical strains is alarming and reflects overuse of carbapenems. Hence, early detection and prompt initiation of infection control practices are vital to mitigate the further spread of MBLs. Additionally, it is also important to follow antimicrobial prescription guidelines toavoidtheinappropriateuse ofcarbapenems.

The limitation of this study is that the genotypic tests could not be performed for the MBL producing isolates due to resource constrains. Also, the study duration was shorter.

## CONCLUSIONS

In conclusion, we found a high percentage of MBL producing isolates among Acinetobacter baumannii and Pseudomonas aeruginosa isolates. With the increasing use of carbapenems in the treatment of critically ill patients, the problems of MBL production are also expanding at an alarming rate. Therefore, it is mandatory to perform routine monitoring of MBL producing isolates in clinical laboratories in order to help the clinicians prescribe proper antibiotics.

## References

[1] Palzkill T (2013). Metallo-3-lactamase structure and function.. Ann N Y Acad Sci..

[2] Potron A, Poirel L, Nordmann P (2015). Emerging broad-spectrum resistance in Pseudomonas aeruginosa and Acinetobacter baumannii: Mechanisms and epidemiology.. Int J Antimicrob Agents..

[3] Kazmierczak KM, Rabine S, Hackel M, McLaughlin RE, Biedenbach DJ, Bouchillon SK, Sahm DF, Bradford PA (2015). Multiyear, Multinational Survey of the Incidence and Global Distribution of Metallo-β-Lactamase-Producing Enterobacteriaceae and Pseudomonas aeruginosa.. Antimicrob Agents Chemother..

[4] Hong DJ, Bae IK, Jang IH, Jeong SH, Kang HK, Lee K (2015). Epidemiology and Characteristics of Metallo-ß-Lactamase-Producing Pseudomonas aeruginosa.. Infect Chemother..

[5] Shrestha S, Amatya R, Adhikari RP (2015). Prevalence and antibiogram of Pseudomonas aeruginosa isolated from clinical specimens in a Teaching Hospital, Kathmandu.. Nepal Med Col! J..

[6] Thapa P, Bhandari D, Shrestha D, Parajuli H, Chaudhary P, Amatya J, Amatya R (2017). A hospital based surveillance of metallo-beta-lactamase producing gram negative bacteria in Nepal by imipenem-EDTA disk method.. BMC Res Notes..

[7] Winn W, Allen S, Janda W (2006). Koneman's color atlas and textbook of diagnostic microbiology..

[8] CLSI. (2016). M100S. Clinical and Laboratory Standard Institute. Performance standard for antimicrobial disk susceptibility tests..

[9] Bauer AW, Kirby WM, Sherris JC, Turck M (1966). Antibiotic susceptibility testing by a standardized single disk method.. Am J Clin Pathol..

[10] Magiorakos AP, Srinivasan A, Carey RB, Carmeli Y, Falagas ME, Giske CG (2012). Multidrug-resistant, extensively drug-resistant and pandrug-resistant bacteria: an international expert proposal for interim standard definitions for acquired resistance.. Clin Microbiol Infect..

[11] Shivaprasad A, Antony B, Shenoy P (2014). Comparative evaluation of four phenotypic tests for detection of Metallo-ß-lactamase and Carbapenemase production in Acinetobacter baumannii.. J Clin Diagnostic Res..

[12] Lee K, Lim YS, Yong D, Yum JH, Chong Y (2003). Evaluation of the Hodge test and the imipenem-EDTA double-disk synergy test for differentiating metallo-ß-lactamase-producing isolates of Pseudomonas spp. and Acinetobacter spp.. J Clin Microbiol..

[13] Yong D, Lee K, Yum JH, Shin HB, Rossolini GM, Chong Y (2002). Imipenem-EDTA disk method for differentiation of metallo-beta-lactamase-producing clinical isolates of Pseudomonas spp. and Acinetobacter spp.. J Clin Microbiol..

[14] Franklin C, Liolios L, Peleg AY (2006). Phenotypic detection of carbapenem-susceptible metallo-ß-lactamase-producing gram-negative bacilli in the clinical laboratory.. J Clin Microbiol..

[15] Peymani A, Nahaei MR, Farajnia S, Hasani A, Mirsalehian A, Sohrabi N, Abbasi L (2011). High prevalence of metallo-beta-lac-tamase-producing acinetobacter baumannii in a teaching hospital in Tabriz, Iran.. Jpn J Infect Dis..

[16] Jahan N, Khatoon R, Rashid M (2018). Phenotypic Evaluation of Prevalence of Metallo-Beta-Lactamase (MBL) Production among Clinical Isolates of Pseudomonas aeruginosa and Acinetobacter Species in a Tertiary Care Hospital of North India.. Int J Curr Microbiol App Sci..

[17] Azimi A, Peymani A, Pour PK (2018). Phenotypic and molecular detection of metallo-ß-lactamase-producing Pseudomonas aeruginosa isolates from patients with burns in Tehran, Iran.. Rev Soc Bras Med Trop..

[18] Kazi M, Nikam C, Shetty A, Rodrigues C (2015). Dual-tubed multiplex-PCR for molecular characterization of carbapenemases isolated among Acinetobacter spp. and Pseudomonas spp.. J Appl Microbiol..

[19] Pitout JD, Gregson DB, Poirel L, McClure JA, Le P, Church DL (2005). Detection of Pseudomonas aeruginosa producing metallo-beta-lactamases in a large centralized laboratory.. J Clin Microbiol..

[20] John S, Balagurunathan R (2011). Metallo beta lactamase producing Pseudomonas aeruginosa and Acinetobacter baumannii.. Indian J Med Microbiol..

[21] Dumaru R, Baral R, Shrestha LB (2019). Study of biofilm formation and antibiotic resistance pattern of gram-negative Bacilli among the clinical isolates at BPKIHS, Dharan.. BMC Res Notes..

[22] De AS, Kumar SH, Baveja SM (2010). Prevalence of metallo-ß-lactamase producing Pseudomonas aeruginosa and Acinetobacter species in intensive care areas in a tertiary care hospital.. Indian J Crit Care Med..

[23] Gautam S, Bhattarai NR, Rai K, Poudyal A, Khanal B (2020). Detection of bla NDM-1 Encoding Imepenemase among the Imipenem-Resistant Gram-Negative Bacilli Isolated from Various Clinical Samples at a Tertiary Care Hospital of Eastern Nepal: A Descriptive Cross-Sectional Study.. Int J Microbiol..

[24] Kaur A, Singh S (2018). Prevalence of Extended Spectrum Betalactamase (ESBL) and Metallobetalactamase (MBL) Producing Pseudomonas aeruginosa and Acinetobacter baumannii Isolated from Various Clinical Samples.. J Pathog.

[25] Baniya B, Pant ND, Neupane S (2017). Biofilm and metallo beta-lactamase production among the strains of Pseudomonas aeruginosa and Acinetobacter spp. at a tertiary care hospital in Kathmandu, Nepal.. Ann Clin Microbiol Antimicrob..

[26] Irfan S, Zafar A, Guhar D, Ahsan T, Hasan R (2008). Metallo-ß-lac-tamase-producing clinical isolates of Acinetobacter species and Pseudomonas aeruginosa from intensive care unit patients of a tertiary care hospital.. Indian J Med Microbiol..

[27] Koirala A, Agrahari G, Dahal N, Ghimire P, Rijal KR (2017). ESBL and MBL mediated resistance in clinical isolates of non-fermenting gram negative bacilli (NFGNB) in Nepal.. J Microbiol Antimicrob agents..

[28] Alkasaby NM, El Sayed Zaki M (2017). Molecular Study of Acinetobacter baumannii Isolates for Metallo-P-Lactamases and Extended-Spectrum-P-Lactamases Genes in Intensive Care Unit, Mansoura University Hospital, Egypt.. Int J Microbiol..

[29] Anane YA, Apalata T, Vasaikar S, Okuthe GE, Songca S (2020). Molecular Detection of Carbapenemase-Encoding Genes in Multidrug-Resistant Acinetobacter baumannii Clinical Isolates in South Africa.. Int J Microbiol.

[30] Mishra SK, Acharya J, Kattel K, Koirala J, Rijal BP, Pokhrel BM (2012). Metallo-beta-lactamase producing gram-negative bacterial isolates.. J Nepal Health Res Counc..

[31] Motbainor H, Bereded F, Mulu W (2020). Multi-drug resistance of blood stream, urinary tract and surgical site nosocomial infections of Acinetobacter baumannii and Pseudomonas aeruginosa among patients hospitalized at Felegehiwot referral hospital, Northwest Ethiopia: a cross-sectional study.. BMC Infect Dis..

[32] Bhandari P, Thapa G, Pokhrel BM, Bhatta DR, Devkota U (2015). Nosocomial Isolates and Their Drug Resistant Pattern in ICU Patients at National Institute of Neurological and Allied Sciences, Nepal.. Int J Microbiol..

[33] Anwar M, Ejaz H, Zafar A, Hamid H (2016). Phenotypic Detection of Metallo-Beta-Lactamases in Carbapenem Resistant Acinetobacter baumannii Isolated from Pediatric Patients in Pakistan.. J Pathog..

[34] Kaur A, Gupta V, Chhina D (2014). Prevalence of metallo-ß-lactamase-producing (MBL) Acinetobacter species in a tertiary care hospital.. Iran J Microbiol..

[35] Acharya A, Gurung R, Khanal B, Ghimire A (2010). Bacteriology and antibiotic susceptibility pattern of peritonsillar abscess.. JNMA J Nepal Med Assoc..

[36] Shrestha LB, Baral R, Poudel P, Khanal B (2019). Clinical, etiological and antimicrobial susceptibility profile of pediatric urinary tract infections in a tertiary care hospital of Nepal.. BMC Pediatr..

